# Comparison of an Addictive Potential of μ-Opioid Receptor Agonists with G Protein Bias: Behavioral and Molecular Modeling Studies

**DOI:** 10.3390/pharmaceutics14010055

**Published:** 2021-12-27

**Authors:** Lucja Kudla, Ryszard Bugno, Sabina Podlewska, Lukasz Szumiec, Lucja Wiktorowska, Andrzej J. Bojarski, Ryszard Przewlocki

**Affiliations:** 1 Department of Molecular Neuropharmacology, Maj Institute of Pharmacology, Polish Academy of Sciences, Smętna 12 Street, 31-343 Krakow, Poland; kudla@if-pan.krakow.pl (L.K.); szumiec@if-pan.krakow.pl (L.S.); wktska@if-pan.krakow.pl (L.W.); 2 Department of Medicinal Chemistry, Maj Institute of Pharmacology, Polish Academy of Sciences, Smętna 12 Street, 31-343 Krakow, Poland; bugno@if-pan.krakow.pl (R.B.); smusz@if-pan.krakow.pl (S.P.); bojarski@if-pan.krakow.pl (A.J.B.)

**Keywords:** G protein-biased μ-opioid receptor agonists, addictive behaviors, molecular modeling

## Abstract

Among different approaches to the search for novel—safer and less addictive—opioid analgesics, biased agonism has received the most attention in recent years. Some μ-opioid receptor agonists with G protein bias, including SR compounds, were proposed to induce diminished side effects. However, in many aspects, behavioral effects of those compounds, as well as the mechanisms underlying differences in their action, remain unexplored. Here, we aimed to evaluate the effects of SR-14968 and SR-17018, highly G protein-biased opioid agonists, on antinociception, motor activity and addiction-like behaviors in C57BL/6J mice. The obtained results showed that the compounds induce strong and dose-dependent antinociception. SR-14968 causes high, and SR-17018 much lower, locomotor activity. Both agonists develop reward-associated behavior and physical dependence. The compounds also cause antinociceptive tolerance, however, developing more slowly when compared to morphine. Interestingly, SR compounds, in particular SR-17018, slow down the development of antinociceptive tolerance to morphine and inhibit some symptoms of morphine withdrawal. Therefore, our results indicate that SR agonists possess rewarding and addictive properties, but can positively modulate some symptoms of morphine dependence. Next, we have compared behavioral effects of SR-compounds and PZM21 and searched for a relationship to the substantial differences in molecular interactions that these compounds form with the µ-opioid receptor.

## 1. Introduction

The search for and development of novel opioid agonists have been recently intensified by the current opioid epidemic, which represents a major worldwide public health crisis. It has been shown that certain opioid agonists may preferentially activate one intracellular signaling pathway over another [1]. This concept, called biased agonism or functional selectivity, has become one of the most promising approaches for discovering novel opioid analgesics [2,3]. Because it was suggested that the recruitment of β-arrestin2 (β-arr2) contributes to the adverse effects of morphine [4,5], the idea of compounds presenting G protein bias and minimal β-arr2 recruitment was proposed as a chance for the development of safer μ-opioid receptor (μ-OR) agonists. The data also suggested that G protein-biased μ-OR agonists do not cause adverse effects, such as respiratory depression or gastrointestinal dysfunctions [6,7,8]; however, this statement has been questioned in contemporary works [9,10]. Among the recently discovered G protein-biased μ-OR agonists, some of the SR compounds, i.e., SR-14968 and SR-17018, contain a piperidine core structure. These compounds appear to be a very interesting class, because some of them present a high bias factor and an extremely wide therapeutic window allowing for effective antinociception without respiratory depression [11]. To date, several studies regarding characteristics of SR compounds have been published [11,12,13,14], with most focus placed on SR-14968 and SR-17018 as the most promising and highly G protein-biased members of the SR compound family. The compounds showed sufficient analgesia and long persistence in brain tissue [11]. SR-17018 was previously described to display a high bias factor toward G protein over β-arr2 recruitment [11], but more recently it was also suggested that it acts as a partial μ-OR agonist, which may contribute to an improved therapeutic window [10]. This opioid agonist was shown to induce lower hot plate tolerance than morphine and oxycodone and restore the antinociceptive effectiveness of morphine in animals with developed tolerance [13]. Moreover, it caused abstinence-induced withdrawal symptoms and, at the same time, reduced the symptoms of morphine withdrawal. In other work, the authors showed that SR-17018 induces tolerance in the water tail immersion test, but it is effective in mouse pain models [12]. On the other hand, SR-14968 was tested for the discriminative stimulus effects and produced a fentanyl-like stimulus effect; however, these effects were lower than expected [14].

Although the above-mentioned studies shed light on the general characteristics of SR agonists, the addictive potential of those compounds is still not fully explored. SR-14968 and SR-17018 were not tested in the same experimental models; therefore, the comparison of their effects is difficult.

**Figure 1:**
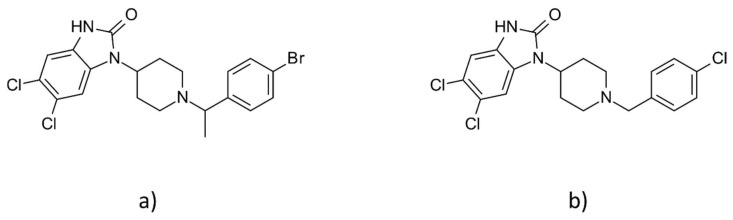
Chemical structures of (**a**) SR-14968 and (**b**) SR-17018.

Here, we performed experiments allowing evaluation of the effects of SR-14968 and SR-17018 (Figure 1) on physiological and subjective aspects of addictive behavior as well as on some symptoms of morphine dependence. We further compared the effects of SR compounds with addictive-like properties of PZM21, another G protein-biased μ-OR agonist [15], characterized in previous work [16]. We found that SR-14968 and SR-17018 possess addictive characteristics, as they are rewarding and cause withdrawal syndrome. Interestingly, the compounds, especially SR-17018, can slightly delay the development of morphine tolerance and attenuate withdrawal in mice dependent on morphine. Our additional aim was to look for potential mechanisms underlying differences between SR-14968, SR-17018 and PZM21 [15] using molecular modeling. The obtained data allowed us to conclude that SR compounds and PZM21 present a different profile of addiction-related symptoms. We also provided molecular modeling data that may indicate putative activity pathways of these compounds and suggest potential structural determinants that may be involved in different intracellular signaling.

## 2. Materials and Methods

### 2.1. Animals

All experiments were performed according to the European Union regulations and the Directive 2010/63/EU and were approved by the II Local Bioethics Committee in Krakow (permit numbers: 261/2019, 31/2020, 351/2020, 196/2021). For all behavioral tests, C57BL/6J male mice were used. They were aged 8 weeks at the beginning of the experiments and weighed 25–30 g. Mice were group-housed, 10 per cage (265 × 180 × 420 mm, Ehret Labor- und Pharmatechnik GMBH & Co.KG, Starnberg, Germany), under standard room temperature 22 ± 2 °C, humidity 50 ± 5%, and 12/12 h light-dark cycle, with free access to food and water (standard diet, Special Diets Services, England). The starting size of each group was 10; however, some animals were excluded from the analysis, i.e., due to death/premature exclusion from the experiment because of poor health or biased preference during the preconditioning session of the conditioned place preference (CPP) test. The total number of animals as well as their suffering was minimized, according to the 3R principle.

### 2.2. Drugs

SR-14968 and SR-17018 were synthesized according to the previously published procedure (Supplementary Material, File S1) [11]. Morphine (Pharma Cosmetic, Warszawa, Poland) was used as a control as well as to study the effects of SR compounds on morphine antinociception, tolerance and dependence. All drugs were prepared in a vehicle (1% DMSO, 10% Kolliphor EL (Sigma Aldrich, St. Louis, MO, USA), 89% dH2O). Drug solutions were freshly prepared from powder and vortexed before each series of injections. In the experiments in which morphine was used, it was dissolved in the same vehicle as SR agonists. For precipitated withdrawal measurement, mice were injected with a non-selective opioid antagonist, naloxone (Merck, Warszawa, Poland). Drugs were administered intraperitoneally (i.p.), only in the experiment assessing the impact of SR compounds on morphine dependence; morphine was administered subcutaneously (s.c.). SR-14698 was administered to mice at doses of 0.3, 1 or 3 mg/kg; SR-17018 was administered at doses of 8, 24, or 48 mg/kg; and morphine was administered at doses of 5 or 10 mg/kg, depending on the experimental schedule.

### 2.3. Antinociception Assessment

Antinociception to thermal nociceptive stimuli was measured using tail flick and hot plate tests as described in more detail in our previous work [16]. Basal responses to nociceptive stimuli were assessed before drug injections.

In the tail flick test (performed using a tail flick apparatus, Ugo Basile, Italy), the light beam was applied to the dorsal side of a mouse’s tail, and the time latency to tail withdrawal or shaking was recorded. To avoid tissue damage, a cut-off latency was set at 9 s.

The hot plate test was carried out using a hot/cold plate apparatus (Ugo Basile, Italy). Mice were placed on a plate heated to 52.5 °C, and the time latency to the first sign of spinally mediated withdrawal reflexes (a paw flinching) as well the first sign of complex behavior (licking/biting of the paw and/or jumping) were measured. A maximum exposure time was set at 30 s (cut-off) to avoid tissue damage. Both responses were expressed as % MPE calculated according to the formula: [(T1 − T0)/(T2 − T0)] × 100, where T0 and T1 are the response latencies before and after drug injection, respectively, and T2 is the cut-off time.

To study the effects of SR agonists on morphine-induced antinociception, mice were pretreated with SR compounds 30 min prior to morphine, and then antinociception was measured using the tail flick test.

### 2.4. Tolerance to Antinociception

To assess the development of tolerance to antinociception induced by SR agonists, mice received injections with the drugs for 7 consecutive days and were tested in the tail flick assay 1 h after drug administration.

In another experiment, aimed to test the influence of SR compounds on the development of tolerance to morphine antinociception, mice were pretreated with SR compounds 30 min prior to morphine, and then the tail flick test was performed.

### 2.5. Physical Dependence and Naloxone-Precipitated Withdrawal

Physical dependence on the SR compounds was measured using a paradigm described previously [16]. Mice were treated with the drugs for 5 consecutive days (2 injections per day at 8:00 and 16:00). Then, on the 6th day, 3 h after the final drug administration, they received naloxone (4 mg/kg) and were placed individually in transparent acryl cylinders (20 cm in diameter, 50 cm in height) for 15 min to observe withdrawal signs, including number of jumps, rearings, paw tremors, wet-dog shakes, teeth chattering episodes and defecations.

In the second experiment, the influence of SR compounds on physical dependence to morphine was tested. To induce dependence, mice were chronically treated with increasing doses of morphine for 4 days as previously reported [17]. Briefly, the procedure of chronic morphine treatment included 3 injections per day (at 8:00, 12:00 and 16:00). The doses used were: day 1: 10 mg/kg, day 2: 20 mg/kg, days 3 and 4: 40 mg/kg. On day 5, 3 h after the final morphine injection (at the dose of 40 mg/kg), they received naloxone and were placed individually in cylinders for 15 min to observe withdrawal symptoms.

The severity of withdrawal was presented as a number of jumps considered as the most prominent symptoms as well as the overall withdrawal score calculated according to the formula described in detail previously [18].

### 2.6. Conditioned Place Preference (CPP) Test

The CPP test was performed as described in our earlier work [16]. Briefly, the CPP apparatus (Med Associates, Fairfax, VT, USA) consisted of 3 different compartments. The procedure began on day 0 with 5 min of acclimatization to the apparatus. On day 1 (preconditioning test), mice were allowed to freely explore the whole apparatus for 20 min, and time spent in each compartment was measured. No significant differences in compartment preference were found within each group during the preconditioning test. During the conditioning period (days 2–11), mice were injected with the drugs (days 3, 5, 7, 9, and 11) or vehicle (days 2, 4, 6, 8, and 10) and immediately placed in the respective compartment for 60 min. The postconditioning test was performed on day 12 and was the same as the preconditioning one. The difference between the times spent in the drug- and vehicle-paired compartments during the postconditioning session was considered to be a measure of CPP (CPP score).

### 2.7. Locomotor Activity

The measurement of locomotor activity lasted for 6 days and was performed using activity chambers (custom-made). Each day, all animals were placed in a chamber for 30 min, and after this time, they received the appropriate drug injection and were placed back in the boxes for an additional 2 h. The expression of locomotor activity was tested after 8 days—the incubation period on day 14. All animals were habituated to the chambers for 2 h, 1 day before the onset of the experiments.

### 2.8. Molecular Modeling

All examined compounds, PZM21, SR-17018, and SR-14968 (both isomers), were docked to the crystal structures of the µ-opioid receptor of the following PDB codes: 4DKL, 5C1M, 6DDF. The compounds were prepared for docking with the use of LigPrep [19] from the Schrödinger Suite; protonation states were generated at pH 7.4 ± 0.0. The proteins were prepared for docking with the use of the Protein Preparation Wizard [20] from the Schrödinger Suite. The docking was carried out in Glide [21] in extra precision mode; the co-crystallized water was not taken into account. The obtained ligand-receptor complexes with the best docking score constituted an input for molecular dynamics (MD) simulations. They were carried out in Desmond [22], using the TIP3P solvent model [23], POPC (palmitoyl-oleil-phosphatidylcoline) as a membrane model, the OPLS3e force-field under the pressure of 1.01325 bar, and a temperature of 300 K. The box shape was orthorhombic with a size of 10 Å × 10 Å × 10 Å. In each case, the system was neutralized by the addition of the appropriate number of Cl- ions and relaxed before simulation; the duration of each simulation was equal to 2000 ns. In addition, on the basis of the ligand-protein complexes obtained in docking, the binding free energy was determined using the Schrödinger’s Prime MM-GBSA tool.

### 2.9. Data Analysis

**Figure 2:**
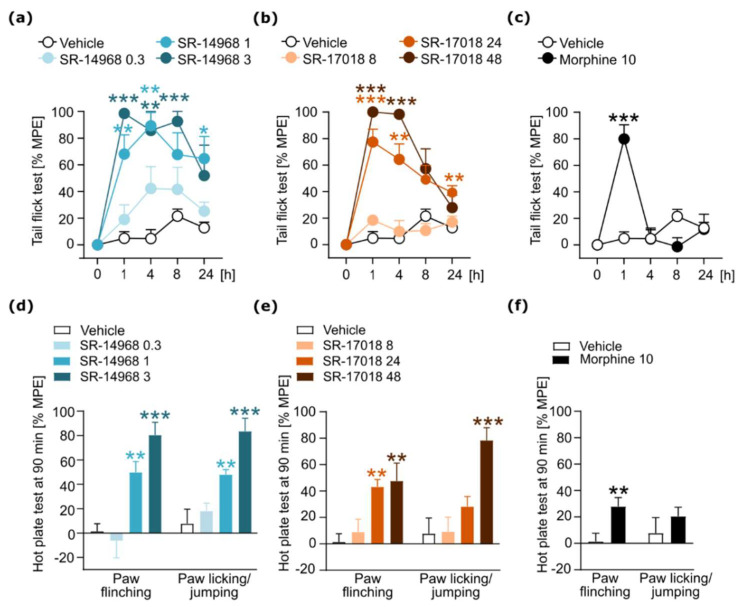
Antinociceptive effects of SR compounds in thermal nociceptive tests. (**a**) Administration of SR-14968 (0.3–3 mg/kg, i.p.) and (**b**) SR-17018 (8–48 mg/kg, i.p.) resulted in an attenuated sensitivity to painful stimulus in the tail flick test. (**c**) Administration of morphine (10 mg/kg, i.p.), used as a positive control, also caused antinociception in the tail flick test. (**d**) Treatment with SR-14968 and (**e**) SR-17018 resulted in antinociceptive effects in the hot plate test. In this test, two types of reactions were assessed: paw flinching and paw licking/jumping. Those reactions were analyzed separately. (**f**) Morphine induced antinociception only to paw flinching behavior. Data are presented as the mean ± SEM. Significant effects of treatment compared with appropriate controls (vehicle group) resulting from post hoc analysis/t-tests are marked with * *p* < 0.05, ** *p* < 0.01, *** *p* < 0.001. Numbers of animals: vehicle *n* = 10; morphine *n* = 10; SR-1468: 0.3 mg/kg *n* = 10, 1 mg/kg *n* = 10, 3 mg/kg *n* = 7; SR-17018: 8 mg/kg *n* = 9, 24 mg/kg *n* = 10, 48 mg/kg *n* = 10.

**Figure 3:**
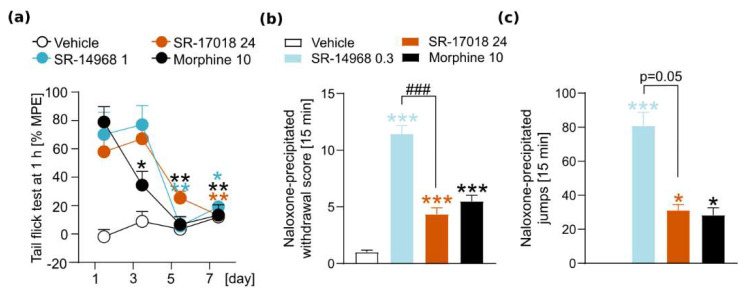
Effects of SR compounds on antinociceptive tolerance and physical dependence. (**a**) Repeated administration of SR-14968 (1 mg/kg, i.p.), SR-17018 (24 mg/kg, i.p.) and morphine (10 mg/kg, i.p.) resulted in the development of antinociceptive tolerance in the tail flick test. However, this effect developed slower in SR compound-treated groups. (**b**) Chronic treatment with all the drugs (SR-14968 at 0.3 mg/kg, SR-17018 at 24 mg/kg and morphine at 10 mg/kg, all drugs injected i.p.) resulted in withdrawal symptoms precipitated by naloxone (4 mg/kg, i.p.), measured as a global withdrawal score. Withdrawal score in SR-14968 group was significantly higher than in group treated with SR-17018. (**c**) In all of the groups, jumping behavior was enhanced. Data are presented as the mean ± SEM. Significant effects resulting from post hoc analysis are marked with * on the graphs: in (**a**) within-group effects significantly different from the first day of the experiment, in (**b**,**c**) effects compared to a vehicle group; differences between SR-agonists-treated groups marked with #. * *p* < 0.05, ** *p* < 0.01, ***/### *p* < 0.001. Numbers of animals: (**a**) vehicle *n* = 10; morphine *n* = 10; SR-1468 *n* = 9; SR-17018 *n* = 10, (**b**,**c**) vehicle *n* = 10; morphine *n* = 10; SR-14968 *n* = 10; SR-17018 *n* = 9.

**Figure 4:**
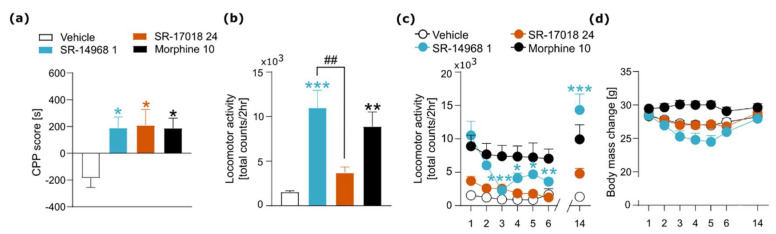
Influence of SR compounds on reward-associated behavior and locomotor activity. (**a**) SR-14968 (1 mg/kg, i.p.), SR-17018 (24 mg/kg, i.p.) and morphine (10 mg/kg, i.p.) induced a preference towards the drug-associated compartment in a CPP test. (**b**) An acute treatment with SR-14968 and morphine, but not with SR-17018, caused an enhancement of locomotor activity. (**c**) Repeated administration of SR-14968 led to the tolerance of locomotor effects, whereas SR-17018- and morphine-treated mice did not present significant changes over a 6-day period of chronic drug treatment. Administration of SR-14968 after the incubation period of 8 days resulted in high expression of locomotor activity on day 14. (**d**) Body mass changes across the experiment. Data are presented as the mean ± SEM. Significant effects resulting from post hoc analysis are marked on the graphs: in (**a**,**b**) effects of treatments compared to a vehicle group are marked with * and differences between SR-agonist-treated groups are marked with #, in (**c**,**d**) with * are marked within-group effects compared to the first day of the experiment and expression of locomotor activity (within-group differences between the activity on day 6 and 14). * *p* < 0.05, **/## *p* < 0.01, *** *p* < 0.001. Numbers of animals: (**a**) vehicle *n* = 9; morphine *n* = 8; SR-1468 and SR-17018 *n* = 10, (**b**) all groups *n* = 10, (**c**,**d**) vehicle *n* = 10; morphine *n* = 10; SR-1468 *n* = 9; SR-17018 *n* = 10.

**Figure 5:**
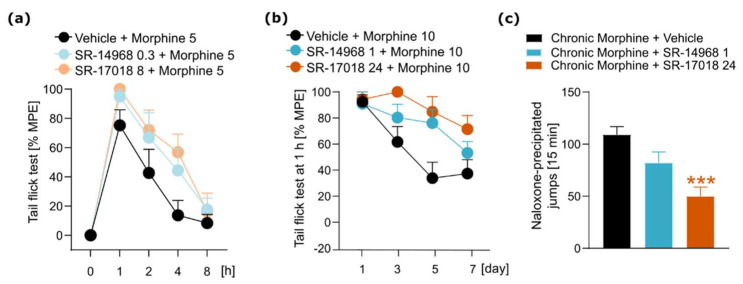
Impact of SR compounds on behavioral effects of morphine. (**a**) Administration of SR-14968 (0.3 mg/kg, i.p.) and SR-17018 (8 mg/kg, i.p.) 30 min prior to morphine (5 mg/kg, i.p.) slightly enhanced morphine-induced antinociception. (**b**) Pretreatment with SR-14968 (1 mg/kg, i.p.) and SR-17018 (24 mg/kg, i.p.) 30 min prior to daily morphine administration (10 mg/kg, i.p.) slightly delayed the development of tolerance to morphine-induced antinociception. (**c**) Pretreatment with SR-14968 (1 mg/kg, i.p.) and SR-17018 (24 mg/kg, i.p.) 30 min prior to naloxone (4 mg/kg, i.p.), resulted in a tendency to (SR-14968)/significant decrease (SR-17018) in the number of withdrawal jumps. Data are presented as the mean ± SEM. Significant effects resulting from post hoc analysis are marked with * (comparison to a group cotreated with morphine and vehicle). *** *p* < 0.001. Numbers of animals: (**a**,**c**) all groups *n* = 9, (**b**) all groups *n* = 10.

Statistical analyses were performed using GraphPad Prism (version 8.0, 2018, GraphPad Software, San Diego, CA, USA). For the data analysis, we performed an unpaired two-tailed *t*-test (Figure 2f), a one-way ANOVA (Figure 2d,e, Figure 3b,d, Figure 4a,b and Figure 5b,c), Kruskal–Wallis test (Figure 3c) or two-way repeated-measures ANOVA (Figure 2a–c, Figure 3a, Figure 4c,d and Figure 5a). Post-hoc analysis was performed using Sidak’s, Dunnett’s or Dunn’s multiple comparisons tests. Data are presented in the figures as the mean ± SEMs. Statistically significant differences between tested groups are marked with the symbols * or # (*/# *p* < 0.05; **/## *p* < 0.01; ***/### *p* < 0.001).

## 3. Results

### 3.1. SR Compound-Induced Antinociception

At the beginning of the study, we performed tail flick and hot plate tests to assess the antinociceptive effects of SR-14968 and SR-17018. Treatment with both compounds resulted in long-lasting and dose-dependent antinociception in the tail flick test (SR-14968: interaction effect (treatment × time) F_(12,132)_ = 3.169, *p* = 0.0005, Figure 2a; SR-17018: interaction effect (treatment × time) F_(12,140)_ = 12.36, *p* < 0.0001, Figure 2b). Morphine, used as a positive control, also had an antinociceptive effect in this test (interaction effect (treatment × time) F_(4,72)_ = 18.68, *p* < 0.0001, Figure 2c); however, the duration of morphine-induced antinociception was shorter than that evoked by SR compounds, indicating their different pharmacokinetic properties.

In the hot plate test, we distinguished two types of reactions to nociceptive stimuli: paw flinching and licking of the paw or jumping. Administration of SR compounds increased the time latency to both types of reaction in a dose-dependent manner (SR-14968: paw flinching F_(3,33)_ = 14.51, *p* < 0.0001, paw licking/jumping F_(3,33)_ = 13.86, *p* < 0.0001, Figure 2d; SR-17018: paw flinching F_(3,35)_ = 6.385, *p* = 0.0014, paw licking/jumping F_(3,35)_ = 10.77, *p* < 0.0001, Figure 2e). Treatment with morphine had little effect in this test, probably due to the time point chosen for the measurement (paw flinching t_(18)_ = 2.899, *p* = 0.0096, paw licking/jumping t_(18)_ = 0.9240, *p* = 0.3677, Figure 2f).

Based on the dose-response curves obtained in the tail flick test, we chose the middle doses inducing antinociception at the level of about 70–80% of MPE 1 h after the drug injection for further experiments, which were aimed to assess the effects of SR-14968 and SR-17018 on the development of addiction-like behavior. In a test measuring naloxone-precipitated withdrawal in mice chronically treated with SR compounds, we decided to use the dose of 0.3 mg/kg i.p. of SR-14968. It was based on our previous experience, as we anticipated that a higher dose (1 mg/kg i.p.) of this compound administered twice daily would cause a significant deterioration of animal well-being. The single injection of 0.3 or 1 mg/kg of the SR agonist was used for the assessment of the influence on morphine antinociception and tolerance, respectively, and the effect of 1 mg/kg on the naloxone-precipitated morphine withdrawal (Figure 5).

### 3.2. The Impact of SR Compounds on Antinociceptive Tolerance and Physical Dependence

In the next part of the study, we assessed the physiological symptoms of addiction—tolerance to antinociception and precipitated withdrawal syndrome in mice dependent on SR compounds. The results showed that repeated administration of SR agonists led to tolerance. However, when compared to morphine, it developed more slowly, as morphine-induced tolerance was observed on day 3, while such an effect in SR compound-treated groups was noticed on day 5 or 7 (interaction effect (treatment × time) F_(9,105)_ = 5.463, *p* < 0.0001, Figure 3a).

Chronic treatment with SR compounds resulted also in physical dependence, as measured by naloxone-precipitated withdrawal signs (F_(3,35)_ = 65.99, *p* < 0.0001, Figure 3b). Animals treated with SR-14968 presented robust withdrawal, while those treated with SR-17018 displayed withdrawal signs comparable to those induced by chronic morphine. Interestingly, jumps, considered as the most common withdrawal symptom in mice, were enhanced in both SR compound- and morphine-treated mice; however, again, this behavior was exaggerated in animals from the SR-14968 group (H_(3)_ = 31.33, *p* < 0.0001, Figure 3c). Chronic treatment with the drugs resulted in significant loss of body weight in animals from SR compounds and morphine groups (F_(3,35)_ = 38.18, *p* < 0.0001). Therefore, our results indicate that regardless of their G protein bias, SR compounds, especially SR-14968, induce profound physical dependence in mice.

### 3.3. The Effects of SR Compounds on Reward-Associated Behavior and Locomotor Activity

Next, we determined the influence of SR compounds on subjective aspects of addiction-like behavior. We performed a CPP procedure to assess the impact of SR-14968 and SR-17018 on reward-associated memory. The data obtained indicate that treatment with both SR compounds and morphine had rewarding effects in this test (F_(3,33)_ = 4.266, *p* = 0.0119, Figure 4a).

We then measured animals’ locomotor activity over 6 consecutive days to test whether SR compounds cause sensitization/tolerance of locomotor effects. On the first day of the experiment, the acute effects of the tested compounds on locomotion were assessed. The results showed that morphine- and SR-14968-treated mice presented enhanced locomotor activity when compared to control animals (F_(3,36)_ = 11.29, *p* < 0.0001, Figure 4b). Locomotor activity of mice from the SR-14968 group was significantly higher than in animals from the SR-17018 group, indicating differential effects of SR agonists on locomotion. Data analysis of locomotor activity over the 6-day period showed time-dependent changes in the activity of the studied groups (interaction effect (treatment × time) F_(18,245)_ = 2.583, *p* = 0.0006, Figure 4c). In the group of animals receiving SR-14968, the locomotor activity was tolerated. No tolerance was noted in mice treated with SR-17018 or morphine.

The expression of locomotor activity was measured after an 8-day incubation period. The data show that only the treatment with SR-14968 resulted in a significantly increased expression of locomotor effects (interaction effect (treatment × time) F_(3,70)_ = 6.093, *p* = 0.001, Figure 4c). Hence, in our experiment, we showed slight, but nonsignificant locomotor sensitization following the treatment with SR-17018. A time course of body mass changes across this experiment did not show statistically significant differences (interaction effect (treatment × time) F_(18,245)_ = 1.514, *p* = 0.0853, Figure 4d); however, we could notice a tendency of repeated administration of SR compounds, in particular SR-1468, to result in an initial weight loss and a return to baseline after the incubation period, which may resemble the specific time course of locomotor activity during the whole procedure.

### 3.4. The Influence of SR Compounds on Morphine-Induced Antinociception, Tolerance and Physical Dependence

Finally, we aimed to assess the influence of SR agonists on certain behavioral effects of morphine. We tested the impact of SR compounds on morphine-induced antinociception. Moreover, taking into account that SR compounds present prolonged action and, when compared to morphine, tolerance to their antinociceptive effects develops more slowly, we aimed to test their ability to modulate morphine-induced tolerance and physical dependence. SR agonists or vehicle were administered 30 min prior to morphine injection, and antinociceptive responses were measured using the tail flick test. We used the dose of 5 mg/kg of morphine to evoke a moderate antinociceptive effect and the lowest doses of SR compounds, as those agonists had strong antinociceptive action. There is a tendency toward enhanced antinociception in mice cotreated with SR agonists and morphine (treatment effect F_(2,24)_ = 5.17, *p* = 0.0136); however, the analysis showed no statistically significant interaction effect ((treatment × time) F_(8,96)_ = 0.7646, *p* = 0.6347, Figure 5a).

We then tested the effects of SR agonists on the development of morphine-induced antinociceptive tolerance. The drugs were administered 30 min prior to each morphine injection (days 1–7). The data analysis showed no interaction effect ((treatment × time) F_(6,81)_ = 1.459, *p* = 0.2026, Figure 5b). However, again there was a strong tendency toward the delayed development of morphine tolerance in mice additionally treated with SR compounds (treatment effect F_(2,27)_ = 12.82, *p* = 0.0001).

In the next experiment, we tested the influence of SR agonists on the severity of withdrawal symptoms in mice chronically treated with escalating doses of morphine by administration of SR compounds 30 min prior to naloxone. Pretreatment with SR-17018 resulted in a decrease in the number of jumps (F_(2,24)_ = 10.69, *p* = 0.0005, Figure 5c) and a tendency toward the attenuation of global withdrawal score (F_(2,24)_ = 2.434, *p* = 0.1091). SR-14968 slightly attenuated withdrawal; however, no statistically significant changes were observed.

### 3.5. Molecular Modeling

In our previous work [16], we characterized the behavioral effects of PZM21, another μ-OR agonist proposed to preferentially act via the G protein [15]. As presented in Table 1, we noticed some interesting differences between the effects of PZM21 and SR agonists on nociception and addictive behavior. Thus, we asked about the possible mechanisms underlying them.

At first, interaction of compounds with µ-opioid receptor at different states was examined via docking (4DKL represents inactive receptor form, whereas 5C1M and 6DDF refer to its activated conformation). As SR-14968 was experimentally tested as racemate, both its isomers were modeled independently.

**Figure 6:**
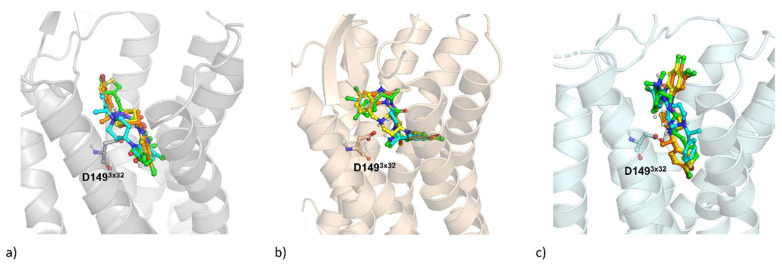
Docking results of PZM-21 (green), (R)-SR-14968 (cyan), (S)-SR-14968 (orange), SR-17018 (yellow) to (**a**) 4DKL, (**b**) 5C1M, and (**c**) 6DDF crystal structures of the µ-opioid receptor. The amino acid numbers presented in superscript follow the GPCRdb numbering scheme [24].

**Figure 7:**
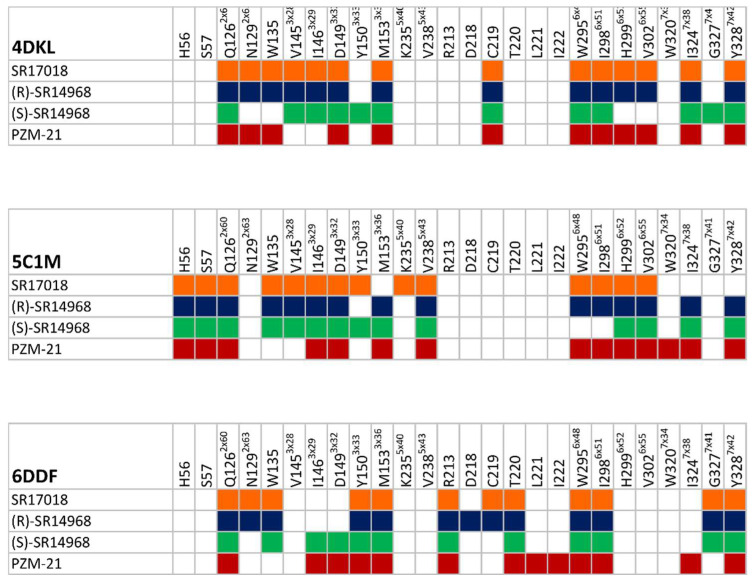
Ligand-protein interaction matrices obtained for the modeled compounds. The amino acid numbers presented in superscript follow the GPCRdb numbering scheme [24].

The obtained docking poses are presented in Figure 6. Ligand-receptor interaction matrices, prepared to facilitate results interpretation, are presented in Figure 7; 2D ligand-protein interaction schemes are placed in the Supplementary Material (Figure S1).

The docking results indicate that although (R)-SR-14968 adopts a slightly different orientation of the piperazine moiety in 4DKL crystal structure, the set of amino acids interacting with this ligand in the receptor binding site is similar to the set of residues, which form contact with SR-17018 and PZM-21. The most visible differences in the ligand-protein interaction matrices referring to the 4DKL-based dockings involve lack of interaction by (S)-SR-14968 with N129^2×63^ and W135, as well as two residues from the 6th transmembrane helix (TM6) and lack of contact by PZM-21 with V145^3×28^ and I146^3×29^.

For 5C1M, which represents an activated form of the µ-opioid receptor, it was SR-17018 that slightly shifted the orientation of the piperidine and chlorophenyl moieties in comparison to PZM-21 and both isomers of SR-14968. It is reflected in the ligand-interaction matrix as a lack of interaction by SR-17018 with TM7. The most consistent poses of all SR agonists examined were obtained in the 6DDF-based dockings, with only slight differences in contact patterns with the second extracellular loop (ELC2). In the case of experiments with 6DDF, it was PZM-21 that adopted a slightly different orientation in comparison to the rest of the compounds, and it was manifested mainly by more frequent interaction by PZM-21 with ELC2.

In addition, for each ligand-receptor complex, the binding free energies were determined (Table 2).

The calculated binding free energies vary depending on the crystal structure used. For the inactive receptor form (4DKL), the highest binding energy was calculated for PZM-21 (−54.41 kcal/mol), and the lowest (−36.77 kcal/mol) for (S)-SR-14968. Interestingly, for this crystal structure, the highest differences between SR-14968 isomers were obtained (~13 kcal/mol in comparison to ~3–4 kcal/mol for 5C1M and 6DDF crystals). SR compounds were characterized by the highest binding energies for 5C1M crystal structure, whereas for 6DDF, it was again PZM21, for which the highest energy was supposed to be released upon binding. It should be remembered that such differences might be a result of slight variations in the conformation of the receptor binding sites for 5C1M and 6DDF, as 5C1M conformation is stabilized by the small molecule agonist BU72, whereas in the 6DDF, peptide agonist DAMGO was co-crystallized.

**Figure 8:**
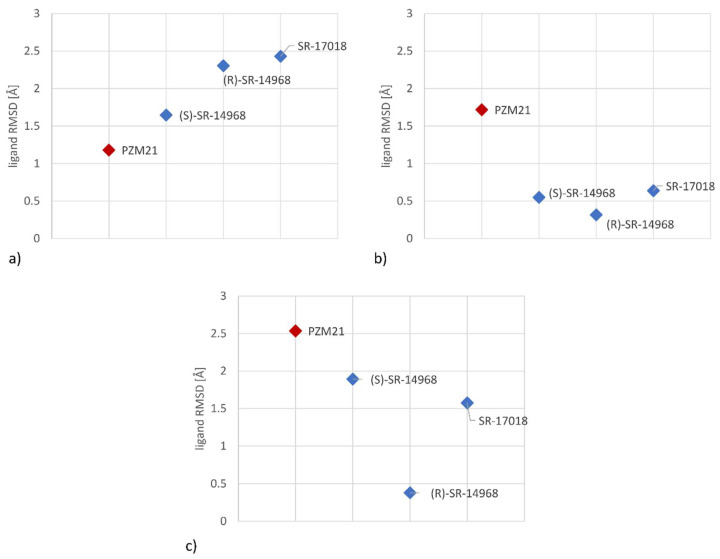
Analysis of the average ligand RMSD during MD simulations for (**a**) 4DKL, (**b**) 5C1M, (**c**) 6DDF crystal structures.

As the set of amino acids differentiating examined compounds varied for different crystal structures used in the study, MD simulations for all of the obtained ligand-receptor complexes were carried out. Ligand-protein interaction diagrams, which enable detailed analysis of changes in the ligand-protein contact patterns, are included in the Supplementary Material (Figure S2). Visual comparison of the positions from the selected frames (Supplementary Material, Figure S3) indicate that there is a relationship between the flexibility of SR compounds and PZM21 in the µ-opioid receptor binding site during MD simulations. In order to examine this effect more formally, the root mean square deviation (RMSD) of ligand atoms was monitored (Figure 8). This parameter informs about the variation of atom positions with reference to its initial state. In molecular dynamics, it measures the change in the displacement of selected atoms for the particular frame with respect to a reference frame. In our study, RMSD was determined for a ligand for each frame, with respect to the first frame, and finally the obtained values were averaged.

The ligand RMSD values clearly indicate that SR compounds (depicted in blue) are more stably fitted in the activated µ-opioid receptor (simulations for 5C1M, 6DDF) in comparison to PZM21 (red). On the other hand, the situation is reversed for the 4DKL crystal structure (which refers to the inactive receptor state) when atoms of PZM21 change their position to a lower extent than observed for SR compounds.

**Figure 9:**
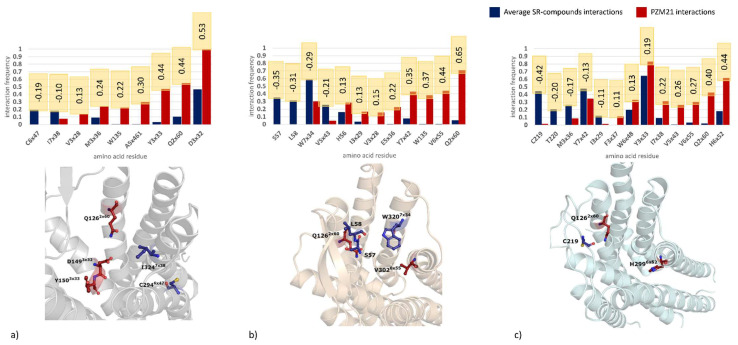
Analysis of residues with the highest discriminative power between the SR compounds and PZM21 for (**a**) 4DKL, (**b**) 5C1M, (**c**) 6DDF crystal structures; numbers in orange boxes refer to differences in the interaction frequencies between PZM21 and SR compounds. Example residues are visualized.

In order to indicate amino acids that might be crucial for specific compound effects observed in behavioral studies, a detailed analysis of the frequency of ligand-receptor interactions during MD simulations was carried out. As we were mainly interested in finding discriminants between the SR compounds and PZM21, all data obtained for SR compounds were averaged and compared with the PZM21 interaction patterns. Differences in the frequencies of interactions during MD between SR compounds and PZM21 were determined for each amino acid of the target proteins, and positions with the highest discriminative power are gathered in Figure 9.

Analysis of the interaction patterns obtained during MD simulations indicated that there is a quite extensive set of positions with significant differences in the contact frequencies between PZM21 and SR compounds. There are positions for which a difference of over 50% in the interaction frequency occurred during MD, such as D149^3×32^ for 4DKL and Q126^2×60^ for 5C1M. Interestingly, Q126^2×60^ is a residue for which such high differences occur consistently for all crystal structures examined. In all cases, it was PZM21 that made contact with this amino acid more frequently in comparison to SR compounds. When only activated receptor forms are considered (5C1M, 6DDF), there are 4 positions with high discriminative SR compound/PZM21 power: V238^5×43^, V302^6×55^, I146^3×29^, and Y328^7×42^; however, in this case, the tendencies in the contact preferences are not clear, as only contact with V302^6×55^ is preferred by PZM21 for both 5C1M and 6DDF. For other positions, the contact preferences varied depending on the crystal structure considered. There is one position (V145^3×28^) that is shared by 4DKL and 5C1M (with consistent contact preferences for PZM21) and 3 residues that are common for the 4DKL and 6DDF pair (Y150^3×33^, M153^3×36^, I324^7×38^), with contact preference consistency occurring only for Y150^3×33^ (more frequent interaction with PZM21).

## 4. Discussion

In the present study, we showed that SR-17018 and SR-14968, highly G protein-biased μ-OR agonists with a wide therapeutic window, possess rewarding properties. Results obtained in the CPP test indicate that both agonists cause reward-associated behavior. It should be mentioned that a vehicle used for the preparation of solutions displayed some aversive properties in the CPP test. As both SR-agonists and morphine were dissolved in the same vehicle, we assume that SR-14968 and SR-17018 induced rewarding effects comparable to morphine. This suggests that G-protein biased μ-OR signaling mediates opioid reward and is in accordance with the results that morphine-induced place preference is enhanced or does not change in mice with disrupted β-arr2 functions [25,26]. On the other hand, previous studies, including our own, have shown that another G-protein biased μ-OR agonist, PZM21, does not induce CPP [15,16] and even causes some aversion at the highest dose tested [16]. However, to some extent, this effect may be explained by the fact that some effects of PZM21 are at least partly mediated by ĸ-OR [15]. Nevertheless, the interdependence between G protein bias and opioid reward should be further investigated.

We also examined whether SR compounds affect the locomotor activity of mice upon both acute and repeated administration. We observed that SR-14968 induces a significant enhancement of acute locomotor activity of mice, but the continuation of treatment with this agonist results in toleration of the locomotor effects (between days 1 and 6). At the same time, no significant changes under both acute and chronic treatment conditions were found in mice from the SR-17018 and morphine groups. After the 8-day incubation period, a profound increase in locomotor activity (about 3-fold greater than in the previous measurement) was noted in animals treated with SR-14968. Again, no significant differences were found in the SR-17018 and morphine groups. Thus, this part of the study revealed a considerable distinction between the effects induced by SR-14968 and SR-17018. In our previous report, we showed that PZM21 did not change animals’ locomotor activity in the same experimental conditions as in the current study [16]. Only in mice treated with the highest dose of PZM21, we observed expression of locomotor activity after the incubation period. However, in comparison with the effects of SR-14968, this expression was very low. Generally, it appears that in terms of influence on locomotor activity, PZM21 and SR-17018 have a lot in common, whereas SR-14968 has the opposite influence on this behavior. Initial results of research obtained in transgenic β-arr2 knockout mice suggested that β-arr2 may be partly responsible for locomotor behavior following morphine administration [26]. Another study demonstrated that morphine-induced increases in locomotor activity require β-arr2 in a dopamine D1 receptor-dependent manner [27]. However, such a role of β-arr2 was not confirmed in a recent study [19]. Our study indicates that the involvement of G protein-biased signaling in opioid-induced locomotor effects is ambiguous, and various G protein-biased compounds may differ in terms of this behavior. Possibly, other signaling mechanisms may play a key role here.

We demonstrated that SR-14968 and SR-17018 exert a dose-dependent analgesia, which is in line with previous observations [11,14]. However, we additionally showed that administration of SR-17018 at a dose of 48 mg/kg was sufficient to evoke the maximal possible antinociceptive response in the tail flick test, suggesting that this compound may act as a full μ-OR agonist. This varies from data provided by Gillis et al. [28], who found that SR-17018 antinociceptive potency was about 50% of the MPE and pointed out the low intrinsic efficacy of this agonist. Secondly, the analgesic effects of the compounds lasted up to 24 h, which may correspond to their prolonged presence in plasma and brain tissue [11]. Therefore, in this respect, SR compounds appear to have an advantage over conventional μ-OR agonists with relatively short action, such as morphine or fentanyl [29].

In the present study, we also showed that repeated treatment with both SR-14968 and SR-17018 results in antinociceptive tolerance. However, the development of tolerance was delayed, when compared to morphine. Previous research was aimed to determine the development of tolerance following chronic SR-17018 administration. Grim et al. [13] reported that this compound does not cause tolerance in the hot plate test upon chronic administration at the dose of 24 mg/kg, which we have also used. Moreover, a recently published study indicates that SR-17018 produces tolerance, as does morphine in the water tail immersion test [12]. Our results indicate the possibility that repeated administration of SR-17018 and SR-14968 induces antinociceptive tolerance, even if it is slightly delayed. The hypothesis regarding the vital role of β-arr2 in antinociceptive tolerance was based on the study by Bohn et al. [5] in which β-arr2 knockout mice failed to develop tolerance to morphine in the hot plate test. Therefore, it is likely that the exclusion of the β-arr2 pathway prevents the hot plate tolerance while having only a moderate effect on the tolerance to spinally mediated reactions measured in the tail flick/water tail immersion tests. The development of tolerance after chronic treatment with SR compounds may depend on the nociceptive stimulus modality, pain condition and type of test used. Alternatively, the potential of a given μ-OR agonist to cause tolerance may be associated with its agonist receptor efficacy, as compounds displaying high intrinsic activity were shown to induce a lower degree of tolerance [30,31,32]. As in our dose-response antinociceptive study both SR compounds behaved as high-efficacy full agonists; delayed development of tolerance may be explained as a result of this pharmacological property. It can also partly justify the observation that PZM21, which is characterized as a partial and low-efficacy μ-OR agonist [16,28,33], induces rapid tolerance [9,16].

The results of the present study indicate that both SR compounds cause physical dependence, as we observed a naloxone-precipitated withdrawal syndrome in mice chronically treated with the compounds. SR-14968-treated mice presented a severe naloxone-precipitated withdrawal, even though this agonist was administered at a very low dose. It can be assumed, therefore, that it is a highly addictive compound. SR-17018 also caused signs of physical dependence, albeit less intense than SR-14968. Grim et al. [13] reported that cessation of SR-17018 administration results in spontaneous withdrawal, which is in line with our results; however, the scheme of treatment, as well as the type of withdrawal, were different. In our previous work, we found that chronic treatment with PZM21 leads to physical dependence, but only at the highest dose tested [16]. Interestingly, withdrawal symptoms in β-arr2 knockout mice were unchanged [5] or only slightly attenuated following a low dose of daily morphine [34]. Lack of direct involvement of β-arr2 in physical dependence and withdrawal syndromes is also supported by results obtained in knock-in mice expressing a μ-OR unable to recruit β-arr2 [19]. Overall, the G protein bias does not appear to affect physical dependence on opioids, and it seems that there is no direct relation between the bias factor and severity of withdrawal. Possibly, a relatively low degree of physical dependence on PZM21 is associated with its other pharmacological features.

Further, our results indicate that SR compounds may influence some morphine-induced effects. SR agonists slightly potentiate morphine-induced antinociception. Co-treatment with morphine and SR agonists may slightly delay the development of tolerance, and pretreatment with SR-17018 can diminish the severity of naloxone-precipitated withdrawal. The results of our study are in line with the data presented by Bohn’s group [13]. The authors showed that substitution with SR-17018 in mice tolerant to morphine restores morphine potency and efficacy in a hot plate test as well as preventing the onset of spontaneous morphine withdrawal. We also observed a tendency toward similar action by SR-14968 in those tests; however, SR-17018 appears as much more effective.

To summarize, we demonstrated that SR-14968 and SR-17018 possess some addictive features. Both compounds induce comparable rewarding effects and cause lightly delayed development of antinociceptive tolerance. SR-14968 leads to more severe naloxone-precipitated withdrawal, indicating that it causes stronger physical dependence. Our observations bring a functional characterization of two agonists from the SR compound group and suggest that high G protein bias may not determine the nonaddictive properties of μ-OR agonists. In the context of the latest controversies regarding the G protein-biased opioids [10,22,35,36], our studies also do not provide conclusive support for their advantage over conventional opioids.

Our data and several other sources of evidence [37] suggest that μ-OR agonists with a high G protein bias, such as SR compounds, induce opioid reward. On the other hand, PZM21 appears to be devoid of morphine-like rewarding and reinforcing activity in rodents [14,15]. However, PZM21 suppresses respiration [9], which seriously limits its possible clinical use. At the same time, SR agonists do not affect respiration [11] in a similar way.

The molecular modeling study did reveal in the μ-OR an important set of positions with significant differences in contact preferences and interactions with PZM21 and SR compounds. There was clear evidence that the stability of the compound orientation in the binding site of the activated form of μ-OR may play a role in the compounds’ intracellular signaling and functional activity. Increased stability of SR compounds in the activated receptor form is in line with the recent findings of Stahl et al. on SR-17018 and structurally related agonists (they were proved to stabilize the receptor in an active state, which remains sensitive to antagonists) [38]. Therefore, further searches for ligands with the SR-like activity profile should be focused on the provision of the stability of their conformation in the respective receptor binding site. Furthermore, due to the high and consistent discriminative power of Q126^2×60^, it is advisable to carefully examine contact with this amino acid during the design of new SR compounds/PZM21 derivatives. This position was also indicated in the recent work by Ricarte et al. [39] (where morphine and fentanyl underwent examination), as the residue, which forms a very strong interaction with fentanyl. Additionally, other positions that were revealed in our studies as discriminative between PZM-21 and SR compounds were mentioned by the paper; D149^3×32^ was indicated in our study for 4DKL-based experiments (PZM-21 preference) and in the work [39], it was assigned to the group of residues with strong interaction with both morphine and fentanyl, C219 (preference for interaction with SR compounds in 6DDF crystal structure in our study; stronger contact with fentanyl when examined by Ricarte et al., and Y150^3×33^ (contact preference with PZM-21; indication of stronger contact with morphine by Ricarte et al.)).

Overall, the available data point out that G protein-biased μ-OR agonists may constitute a heterogeneous group with different opioid adverse effects and addictive potential. The complexity of μ-OR signaling requires an individual assessment of different aspects of behavior, taking into account other pharmacological attributes of the given agonist.

## 5. Conclusions

To conclude, in the current work we showed that SR agonists, despite the G protein bias, still possess some addictive properties, as they induce tolerance and dependence and cause reward-associated behavior. At the same time, our results suggest that these compounds may be useful for the modulation of morphine dependence. The data obtained in studies on SR compounds suggest that G protein signaling appears to mediate reward. It is in contrast to the other G protein-biased opioid, PZM21. Molecular modeling did reveal that there is a set of amino acids of µ opioid receptor with significant differences in the contact preferences between PZM21 and SR compounds, which may suggest that other factors such as downstream effectors should be considered.

## Figures and Tables

**Table 1:** Comparison of the effects of PZM21 and SR agonists on addition-like symptoms.

Behavioral Symptoms	PZM21	SR-14968	SR-17018
Antinociception	Dose-dependent, not reaching the maximum possible effect ^1^	Dose-dependent, reaching the maximum possible effect ^2^	Dose-dependent, reaching the maximum possible effect ^2^
Tolerance	Rapid development ^1^	Slightly delayed development ^2^	Slightly delayed development ^2^
Physical dependence	Yes, but only at the highest dose tested ^1^	Yes ^2^	Yes ^2^
Rewarding effects (conditioned place preference)	No ^1^	Yes ^2^	Yes ^2^
Locomotor activity (acute)	Not changed ^1^	Enhanced ^2^	Not changed ^2^
Locomotor activity (repeated treatment)	Not changed ^1^	Tolerance of locomotor effects ^2^	Not changed ^2^
Expression of locomotor activity after the incubation period	Enhanced at the highest dose tested ^1^	Enhanced ^2^	Not changed ^2^
Tolerance to morphine-induced antinociception	Not changed ^1^	Slightly delayed ^2^	Delayed ^2^
Physical dependence on morphine		Slightly attenuated ^2^	Attenuated ^2^
Respiratory depression	Yes ^3^	Very little ^4^	Very little ^4^

^1^ According to Kudla et al., 2019 [16]. ^2^ Data presented in this manuscript. ^3^ According to Hill et al., 2018 [9]. ^4^ According to Schmid et al., 2017 [11].

**Table 2:** Binding free energies (reported in kcal/mol) of PZM-21 and SR compounds computed on the basis of the ligand-protein complexes obtained in docking.

Crystal Structure/Compound	PZM21	(R)-SR-14968	(S)-SR-14968	SR-17018
4DKL	−54.41	−49.7	−36.77	−44.7
5C1M	−40.82	−60.27	−57.66	−51.3
6DDF	−57.41	−51.96	−48.43	−54.56

## Data Availability

All data are presented within the manuscript and supporting information.

## Supplementary Materials

The following are available online at https://www.mdpi.com/article/10.3390/pharmaceutics14010055/s1. Supplementary material S1. Description of synthesis of compounds SR-14698 and SR-17018. Figure S1. 2D ligand-protein interaction diagrams for complexes obtained in docking. Figure S2. Ligand-protein interaction diagrams obtained during molecular dynamics simulations. Figure S3. Compound orientations obtained for the selected frames of MD simulations: 1st frame: green, 250th frame: cyan, 500th frame: orange, 750th frame: yellow, 1000th frame: red.
