# Supplementary material for: Comparison of an Addictive Potential of μ-Opioid Receptor Agonists with G Protein Bias: Behavioral and Molecular Modeling Studies
Source: Pharmaceutics. 2021 Dec 27;14(1):55. doi: 10.3390/pharmaceutics14010055 (PMC8779292; doi:10.3390/pharmaceutics14010055)
Supplement: Supplementary file 1 [file pharmaceutics-14-00055-s001.zip › pharmaceutics-1507188 SUP for conversion.pdf]

pharmaceutics

Supplementary Materials: Comparison of an Addictive Potential of μ-Opioid Receptor Agonists with G Protein Bias: Behavioral and Molecular Modeling Studies

Lucja Kudla, Ryszard Bugno, Sabina Podlewska, Lukasz Szumiec, Lucja Wiktorowska, Andrzej J. Bojarski and Ryszard Przewlocki

Supplementary Material S1. Description of Synthesis of Compounds SR-14698 and SR-17018

Materials and Methods

Chemistry

Materials. All organic reagents were purchased from Merck and Combi-Blocks and were used without purification. Solvents and inorganic reagents were acquired from Chempur. Reaction progress was monitored by TLC on Merck Silica Gel 60 F 254 on aluminium plates. Column chromatography was performed on Merck Silica Gel 60 (0.063–0.200 mm; 70–230 mesh ASTM).

Analytical methods. UPLC/MS analysis was performed on Waters TQD spectrometer combined with UPLC Acquity H-Class with PDA eLambda detector. Waters Acquity UPLC BEH C18 1.7 μm 2.1 × 50 mm chromatographic column was used, at 40 °C, 0.3 mL/min flow rate and 1.0 μL injection volume (the samples were dissolved in LC-MS grade acetonitrile, typically at a concentration of 0.1–1 mg/mL prior to injection). All mass spectra were recorded under electrospray ionization in positive mode (ESI+) and chromatograms were recorded with UV detection in the range of 190–300 nm. The gradient conditions used were: 80% phase A (water + 0.1% formic acid) and 20% phase B (acetonitrile + 0.1% formic acid) to 100% phase B (acetonitrile + 0.1% formic acid) at 3.0 minutes, kept till 3.5 minutes, then to initial conditions until 4.0 minutes and kept for additional 2.0 minutes. Total time of analysis—6.0 minutes.

^{1}H and ^{13}C NMR spectra were recorded on a Bruker Avance III HD 500 NMR spectrometer. Samples were dissolved in DMSO-d_6 with TMS as the internal standard. The spectral data of the compounds refer to their free bases. Free bases were converted into mesylate salts. Elemental analysis was carried out with UNICUBE® analyzer (Elementar Analysensysteme GmbH).

The synthesis of SR-14968 and SR-17018 were achieved in five-step synthetic routes, according to previously reported methods [1]. In the general procedure, nucleophilic aromatic substitution of a 1,2-dichloro-4-fluoro-5-nitrobenzene starting material with *N*-Boc-4-aminopiperidine was followed by nitro group reduction with Raney nickel catalyst, cyclic urea formation in CDI-mediated coupling reaction, Boc deprotection, and finally reductive amination of 5,6-dichloro-1-(piperidin-4-yl)-2,3-dihydro-1H-1,3-benzodiazol-2-one produced the desired analogues. Reductive amination used 4-chlorobenzaldehyde (SR-17018) or 4- bromoacetophenone (SR-14968).

SR-14968, (±)-5,6-dichloro-1-(1-(1-(4- bromophenyl)ethyl)piperidin-4-yl)-1,3-dihydro-2H-benzo[d]imidazol-2-one methanesulfonate

LC-MS: 100% (Rt = 3.23), ESI(+) m/z found: 469.7 [M+H]$^{+}$. Molecular Weight calc'd for $C_{20}H_{20}BrCl_2N_3O$ = 469.20.

^{1}H NMR (500 MHz, DMSO-d_6) δ ppm: 11.14 (s, 1H), 7.55–7.47 (m, 3H), 7.30 (d, J = 8.0 Hz, 2H), 7.13 (s, 1H), 4.03 (tt, J = 8.4, 5.0 Hz, 1H), 3.53 (p, J = 8.7, 7.7 Hz, 1H), 3.02 (d, J = 11.2 Hz, 1H), 2.84 (d, J = 11.3 Hz, 1H), 2.39–2.21 (m, 2H), 2.09–2.01 (m, 1H), 2.00–1.90 (m, 1H), 1.65 (d, J = 12.0 Hz, 1H), 1.58 (d, J = 11.9 Hz, 1H), 1.30 (d, J = 6.7 Hz, 3H).

^{13}**C NMR** (126 MHz, DMSO-d_6) δ ppm: 153.67, 142.53, 130.93, 129.72, 129.68, 128.35, 122.51, 122.49, 119.70, 109.82, 109.73, 62.46, 51.06, 49.12, 49.08, 28.64, 28.58, 18.67.

Elemental analysis ($C_{20}H_{20}BrCl_2N_3O \cdot CH_4O_3S \cdot 0.25H_2O$): found C: 44.23%, H: 4.42%, N: 7.21%; requires C: 44.26%, H: 4.33%, N: 7.37%.

SR-17018, 5,6-dichloro-1-(1-(4-chlorobenzyl)piperidin-4- yl)-1,3-dihydro-2H-benzo[d]imidazol-2-one methanesulfonate.

LC-MS: 100% (Rt = 3.06), ESI(+) m/z found: 411.7 [M+H]$^+$. Molecular Weight calc'd for $C_{19}H_{18}Cl_3N_3O$ = 410.72.

^{1}**H NMR** (500 MHz, DMSO-d_6) δ ppm: 11.16 (s, 1H), 7.50 (s, 1H), 7.42–7.33 (m, 4H), 7.14 (s, 1H), 4.12 (tt, J = 12.2, 4.2 Hz, 1H), 3.50 (s, 2H), 2.89 (d, J = 11.3 Hz, 2H), 2.34 (qd, J = 12.3, 3.9 Hz, 2H), 2.09 (td, J = 12.0, 2.3 Hz, 2H), 1.67 – 1.60 (m, 2H).

^{13}**C NMR** (126 MHz, DMSO-d_6) δ ppm: 153.68, 137.51, 131.36, 130.52, 129.64, 128.38, 128.11, 122.54, 122.52, 109.86, 109.78, 60.96, 52.31, 50.79, 28.38.

Elemental analysis ($C_{19}H_{18}Cl_3N_3O \cdot CH_4O_3S \cdot 0.5H_2O$): found C: 46.68%, H: 4.58%, N: 8.03%; requires C: 46.57%, H: 4.49%, N: 8.15%.

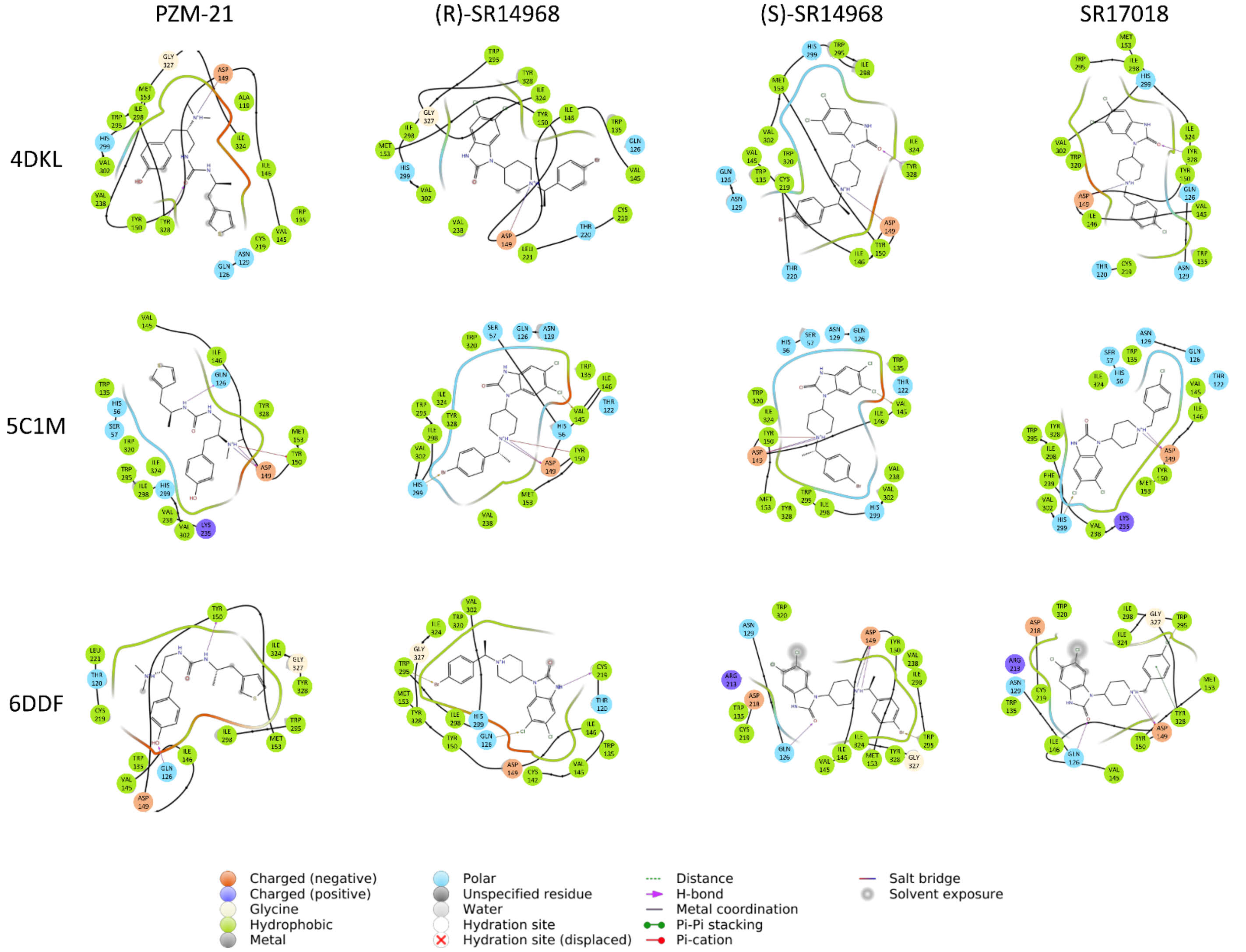

Figure S1. 2D ligand-protein interaction diagrams for complexes obtained in docking.

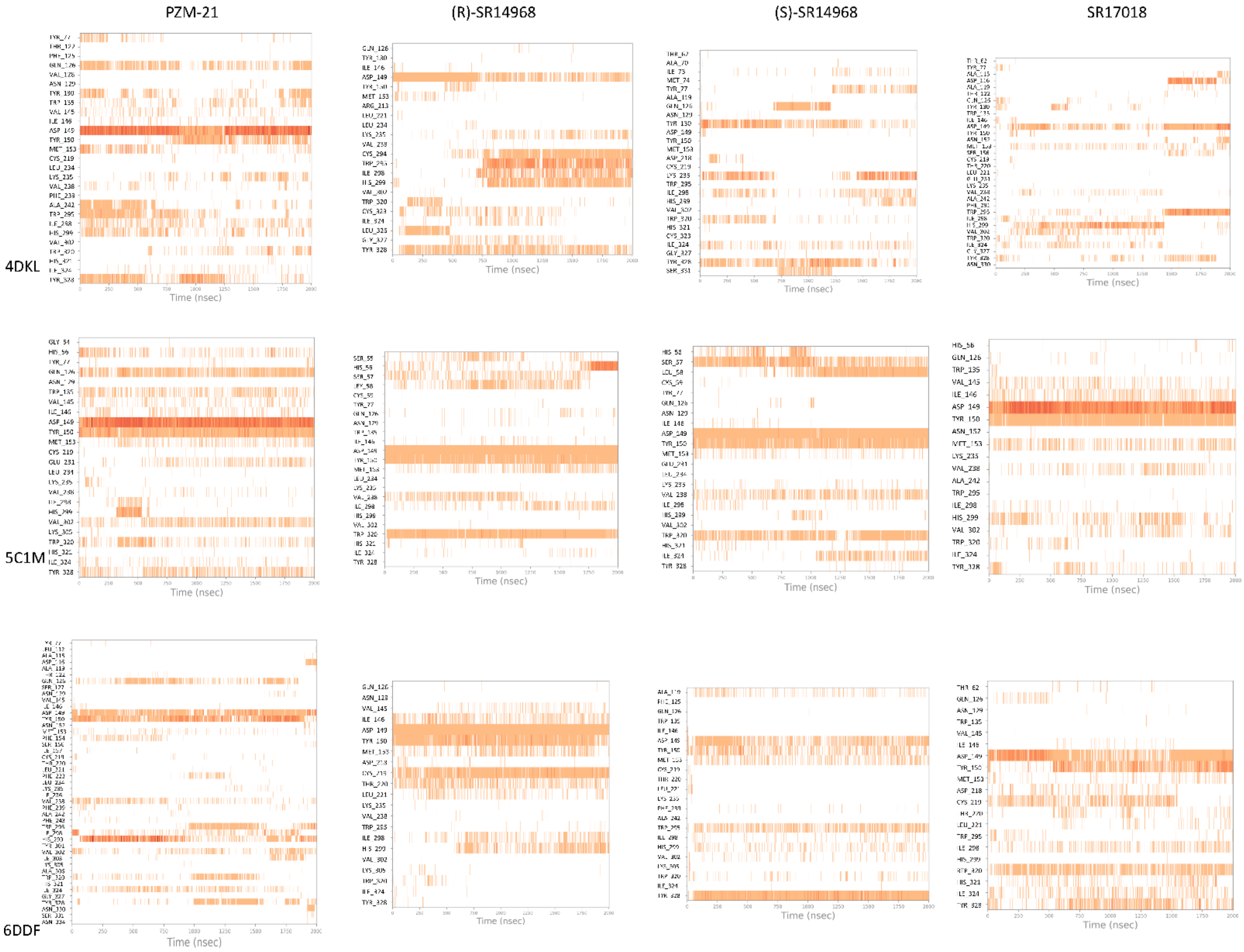

Figure S2. Ligand-protein interaction diagrams obtained during molecular dynamics simulations.

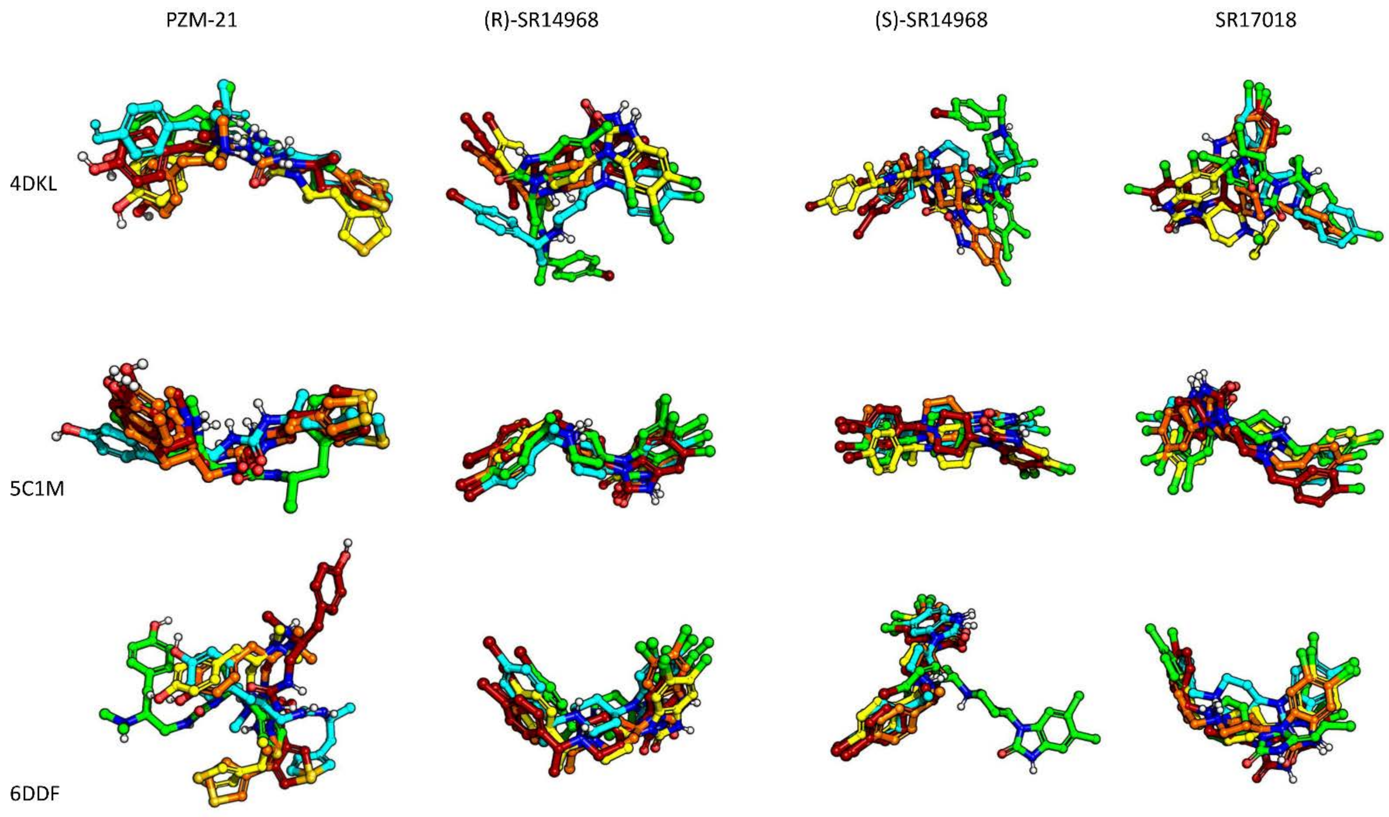

Figure S3. Compound orientations obtained for the selected frames of MD simulations: 1st frame: green, 250th frame: cyan, 500th frame: orange, 750th frame: yellow, 1000th frame: red.

Reference

1. Kennedy, N.M.; Schmid, C.L.; Ross, N.C.; Lovell, K.M.; Yue, Z.; Chen, Y.T.; Cameron, M.D.; Bohn, L.M.; Bannister, T.D. Optimization of a Series of Mu Opioid Receptor (MOR) Agonists with High G Protein Signaling Bias *J. Med. Chem.* **2018**, *61*, 8895–8907.
